# Prediction of SARS-CoV-2 Variant Lineages Using the S1-Encoding Region Sequence Obtained by PacBio Single-Molecule Real-Time Sequencing

**DOI:** 10.3390/v13122544

**Published:** 2021-12-18

**Authors:** Sébastien Lhomme, Justine Latour, Nicolas Jeanne, Pauline Trémeaux, Noémie Ranger, Marion Migueres, Gérald Salin, Cécile Donnadieu, Jacques Izopet

**Affiliations:** 1 Infinity, Université Toulouse, CNRS, INSERM, UPS, 31300 Toulouse, France; Migueres.m@chu-toulouse.fr (M.M.); izopet.j@chu-toulouse.fr (J.I.); 2 Laboratoire de Virologie, CHU Toulouse, Hôpital Purpan, 31300 Toulouse, France; latour.j@chu-toulouse.fr (J.L.); jeanne.n@chu-toulouse.fr (N.J.); tremeaux.p@chu-toulouse.fr (P.T.); ranger.no@chu-toulouse.fr (N.R.); 3 INRAE, US 1426, GeT-PlaGe, Genotoul, 31326 Castanet-Tolosan, France; gerald.salin@inrae.fr (G.S.); cecile.donnadieu@inrae.fr (C.D.)

**Keywords:** SARS-CoV-2, PacBio SMRT sequencing, Illumina sequencing, S1 domain, clade, lineage

## Abstract

The severe acute respiratory syndrome coronavirus 2 (SARS-CoV-2), is the causal agent of the COVID-19 pandemic that emerged in late 2019. The outbreak of variants with mutations in the region encoding the spike protein S1 sub-unit that can make them more resistant to neutralizing or monoclonal antibodies is the main point of the current monitoring. This study examines the feasibility of predicting the variant lineage and monitoring the appearance of reported mutations by sequencing only the region encoding the S1 domain by Pacific Bioscience Single Molecule Real-Time sequencing (PacBio SMRT). Using the PacBio SMRT system, we successfully sequenced 186 of the 200 samples previously sequenced with the Illumina COVIDSeq (whole genome) system. PacBio SMRT detected mutations in the S1 domain that were missed by the COVIDseq system in 27/186 samples (14.5%), due to amplification failure. These missing positions included mutations that are decisive for lineage assignation, such as G142D (*n* = 11), N501Y (*n* = 6), or E484K (*n* = 2). The lineage of 172/186 (92.5%) samples was accurately determined by analyzing the region encoding the S1 domain with a pipeline that uses key positions in S1. Thus, the PacBio SMRT protocol is appropriate for determining virus lineages and detecting key mutations.

## 1. Introduction

The severe acute respiratory syndrome coronavirus 2 (SARS-CoV-2), a member of the *Coronaviridae* family, that caused the COVID-19 pandemic is a ~30 kb single-stranded, positive-sense RNA virus [1,2]. Its genome contains six main open reading frames (ORFs): replicase (ORF1a/ORF1b), spike (S), envelope (E), membrane (M), and nucleocapsid (N), and at least seven other putative ORFs encoding accessory proteins interspersed between the structural genes [3]. To date, a total of 16 proteins were described [4]. The 1273-amino-acid (aa) long S protein and the mature trimeric spike protein is composed of exterior S1 and transmembrane S2 subunits. The S1 subunit attaches the virus to the host receptor (angiotensin converting enzyme 2; ACE2) via its receptor binding domain (RBD, aa residues 319–529) and the S2 subunit ensures fusion of the virus and host cellular membranes [3,5,6,7]. The rate of evolution of SARS-CoV-2 was initially thought to be limited due to the existence of a 3′-5′ exonuclease proofreading function of nonstructural protein 14 (nsp14). However, variants with mutations in the S domain have emerged around the world. One of the first notable variants had a D614G substitution in the S1 domain that increased the affinity of the virus for ACE2 [8]. The SARS-CoV-2 genome has since diverged to produce several clades and lineages that seem to differ in their biology and/or geographic distribution [9]. For instance, the European lineage B.1.177 (clade 20E(EU1)) differs from ancestral sequences at 6 or more positions, including a A222V mutation in the spike protein. This variant arose in Spain early in the summer of 2020 and subsequently spread across Europe, perhaps carried by infected holiday travelers [10]. Another variant belonging to lineage B.1.160 (clade 20A, previously identified 20A.EU2) that has a S477N substitution in the spike protein was common in Autumn 2020 and early 2021 in some European countries, including France [10]. The more recent Alpha variant (lineage B.1.1.7, clade 20I) has spread rapidly from the United Kingdom. It harbors three aa deletions (69del-70del and 144del) and seven mutations in the spike protein, including D614G and N501Y [11]. The emergence of the Beta variant in South Africa (lineage B.1.351, clade 20H) is of great concern as it has since spread worldwide [12]. Variants belonging to this lineage have three mutations in the RBD: K417N, E484K, and N501Y, and several others outside the RBD and are neutralized less efficiently by convalescent and vaccine sera [13,14]. The Gamma variants described in Brazil belonging to the lineage P.1 clade 20J also have the E484K mutation in addition to K417T and N501Y mutations in their RBD [15]. The latest variant of concern, variant Delta (lineage B.1.617.2, clades 21A, 21I and 21J), emerged in India in October 2020 and was later detected in France in May 2021, and has the L452R mutation that makes it more transmissible [16,17]. Since then, many B.1.617.2 sublineages were described (also called AY lineages, for which AY.1 corresponds to B.1.617.2.1) and their number is currently increasing.

The rapid identification of the lineage and related variants that are transmitted more efficiently or that escape the immune response is key for the clinical management of patients, especially those who are immunocompromised that can receive immunotherapies, and monitoring virus spread. Studies demonstrated that certain monoclonal antibodies have low activity on SARS-CoV-2 variants [18,19]. High throughput next-generation sequencing (NGS) methods have been used to study the genomic diversity of SARS-CoV-2 worldwide [20,21,22]. The Pacific Biosciences (PacBio) single-molecule, real-time (SMRT) sequencing system, which records the incorporation of nucleotides into a single DNA template molecule by an immobilized DNA polymerase, provides long and highly accurate sequences thanks to circular consensus reads [23]. It enables full-length virus genome sequencing and a detailed analysis of the spike protein-encoding region [24,25,26].

The aim of this study was to assess the performance of single molecule real time sequencing for both genotyping using the S1 encoding region sequence and determining the haplotypes of the SARS-CoV-2 RNA population thanks to long read sequencing. Compared to Sanger direct sequencing, PacBio sequencing allows high throughput sequencing and determination of the composition of the quasispecies, including low frequency variants. Thus, we compared the results obtained with PacBio SMRT sequencing to those obtained with the Illumina COVIDseq protocol, largely used to define SARS-CoV-2 lineages and clades.

## 2. Materials and Methods

### 2.1. Samples

Nasopharyngeal samples were taken from patients who tested positive for SARS-CoV-2 RNA between 19 January and 26 January 2021 (*n* = 146) and between 10 July and 23 July 2021 (*n* = 54) and stored at −80 °C in the Virology laboratory at Toulouse University Hospital. We selected 200 consecutive samples of these periods for which N gene Ct values were below 25 with the TaqPath™ COVID-19 CE-IVD RT-PCR kit (Thermo Fisher Scientific, Pleasanton, CA, USA) used on the QuantStudio™ 5 Real-Time PCR System (Applied Biosystems, Singapore, Singapore) and that were successfully sequenced with IlluminaCOVIDSeq. They were sequenced on both the PacBio SMRT (S1 region) and Illumina (whole genome) systems.

### 2.2. SARS-CoV-2 RNA Extraction

Virus RNA was extracted from 180 μL transport medium with the MGIEasy Nucleic Acid extraction kit on the MGI SP 960 system (Beijing Genome Institute, Shenzhen, China) according to the manufacturer’s instructions.

### 2.3. PacBio SMRT Sequencing

Primer design: We used two primers from the Pacific Biosciences protocol for full genome sequencing (https://www.pacb.com/wp-content/uploads/Procedure-Checklist-Multiplexing-2.5-kb-Amplicons-for-Whole-Genome-Sequencing-of-SARS-CoV-2.pdf, accessed on 30 June 2020). These primers target a single long amplicon named A6 (2490 nucleotides (nt)) that encodes the full length of the spike protein S1 domain.

cDNA synthesis and library preparation: we generated a single long amplicon named A6 (2490 nucleotides [nt]) that encoded the full length of the spike protein S1 domain. The 25 μL RT reaction mixture contained 10 μL of RNA, 10 µL of water, and 5 μL Superscript IV VILO (Life Technologies, Courtaboeuf, France) and random hexamer oligo(dT) primers. The cycle steps were: 10 min/25 °C, 30 min/50 °C, and 10 min/85 °C.

The A6 cDNA was amplified in 20 μL of reaction mixture: 10 µL of Platinum SuperFi Master Mix, 2 µL of water, 3 µL of forward (A6F; 5′-GTAAAACGACGGCCAGTACAAATCCAATTCAGTTGTCTTCCTATTC-3′) and reverse primer (A6R 5′-CAGGAAACAGCTATGACTGTGTACAAAAACTGCCATATTGCA-3′) M13 tailed primers [27], and 2 µL of cDNA. The cycle steps were: denaturation (98 °C/2 min), amplification (30 cycles of 98 °C/15 s, 65 °C/30 s, 72 °C/2 min), and a final extension (72 °C/5 min).

A second PCR was then performed using barcoding primers tailed with the universal M13 sequence. Reaction mixture: 15 µL of Kapa Ready mix 2× in 9.6 µL of nuclease-free water, 1.7 µL of each barcoded primer (final concentration: 3 µM), and 2 µL of amplified cDNA. Cycling conditions were: 95 °C/5 min, 2 cycles of 98 °C/20 s, 60 °C/15 s, and 72 °C/2 min; then 24 cycles of 98 °C/20 s, 65 °C/15 s, and 72 °C/2 min; final extension, 72 °C/5 min, samples cooled at 12 °C. Products (expected size around 3 kb) were purified with the AMPure PacBio (Pacific Bioscience, Menlo Park, CA, USA) system at ×4.2 according to the manufacturer’s instructions and then quantified with the Quantifluor DSDNA system running on a Roche LC480 instrument according to the manufacturer’s conditions.

Aliquots (10 ng) of each sample were pooled, purified with the AMPure PacBio system (Pacific Bioscience, Menlo Park, CA, USA) at ×0.6, quantified with the Denovix dsDNA BR kit, and the size determined with the Fragment Analyzer system using the kit NGS DNF-473 kit (Agilent, Les Ulis, France), according to the manufacturer’s instructions.

SMRTbell libraries were constructed by pooling 96 barcoded samples. Barcoded amplicon libraries were prepared and sequenced with the SMRTbell Express Template Prep 2.0 kit according to the manufacturer’s instructions. Libraries were quantified at each step with the Qubit HS DSDNA system (Thermo Fisher Scientific, Villebon sur Yvette, France).

Sequencing: samples were prepared with the Sequel binding and internal control kit 3.0 and sequenced with the Sequencing kit by placing 6 pM in the SMRT cell 1M v3 Tray and using 600-min movies on the Sequel Platform (Pacific Bioscience, Menlo Park, CA, USA).

Data analysis: the A6 amplicon consensus sequences were built from the PacBio reads using a home-made Snakemake pipeline. First, the HiFi reads were generated from the PacBio subreads.bam using PacBio CCS tool (v.6.0.0, https://github.com/PacificBiosciences/ccs, accessed on 26 October 2021) and demultiplexed with Lima (v.2.0.0, https://github.com/PacificBiosciences/barcoding, accessed on 26 October 2021). The resulting reads were analyzed by a PacBio open-access tool: pbAA (v.0.1.3, https://github.com/PacificBiosciences/pbAA, accessed on 26 October 2021) which clustered similar reads together and generated one or several haplotypes depending on the diversity of the viral population of each sample. The resulting haplotypes were then mapped on the SARS-CoV-2 reference genome (Wuhan-Hu-1 isolate, Genbank accession number NC_045512.2) with Minimap2 (v2.17) [28] to discard eventual chimeric reads.

In parallel, a consensus sequence was built from the reads and the files were generated by pbAA with another PacBio tool: CoSA (Coronavirus Sequence Analysis, v9.0.0, https://github.com/Magdoll/CoSA, accessed on 26 October 2021). Then, the haplotypes sequences and the consensus sequence (for each sample) were treated separately. Each haplotype/consensus sequence was aligned on the reference SARS-CoV-2 spike region (NC_045512.2) with MAFFT (v.7.475) [29] and trimmed from the beginning of the spike gene (nt 21563) to the end of the A6 amplicon (nt 23823). Aligned sequences were translated into amino acids and again aligned with MAFFT to the spike amino acids reference sequence (YP_009724390.1).

We used a variant table that included the variant strains and their key mutations identified by the French SARS-CoV-2 National Reference Center (NRC) (Table 1). A python script retrieved the mutations (SNP, indel) carried by the haplotype or consensus sequences and compared them to the NRC profiles. A profile was validated if the analyzed sequence harbored 80% of the mutations from the spike region profile covered by the A6 amplicon, as some of the mutations were not present or covered, especially with the Illumina whole genome amplification protocol. The validated profile with the most matching mutations was assigned to the consensus. Conversely, if a sequence did not match the 80% threshold for any profile, it was not assigned (NA).

### 2.4. Illumina COVIDSeq Sequencing

cDNA synthesis and library preparation: cDNA was synthesized from extracted RNA with random hexamers using the ARTIC primers (version 3) and the Illumina COVIDSeq protocol (Illumina, San Diego CA, USA) and amplified using a multiplex PCR protocol that produced 98 amplicons spanning the whole SARS-CoV-2 genome, according to the manufacturer’s instructions. The primer pool also contained primers targeting human RNA that produced an additional 11 amplicons.

Libraries were prepared using the Illumina COVIDSeq protocol (Illumina, USA), according to the manufacturer’s instructions. The PCR-amplified products were later processed for tagmentation and adapter ligation using the IDT for Illumina Nextera UD Indexes Set A, B, C, and D (384 indexes, 384 samples). Samples were further enriched and cleaned up with protocols provided by Illumina. All samples were processed as batches on 96-well plates that included one COVIDSeq positive control HT (CPC HT) and one no-template control (NTC). These 96 libraries were pooled in a microcentrifuge tube and quantified using the Qubit dsDNA HS Assay kit on a Qubit fluorometer (Invitrogen, Villebon sur Yvette, France), according to the manufacturer’s instructions. Fragment sizes were determined in an Agilent Fragment analyzer 5200 (Agilent, Les Ulis, France). The pooled library was normalized to a concentration of 4 nM and 25 μL aliquots of each normalized pool containing index adapter sets A, B, C, and D were combined in a new microcentrifuge tube to produce a final pool of 384 samples at a starting concentration of 4 nM.

Sequencing: the pooled libraries were denatured and neutralized with 0.2N NaOH and 400 mM Tris-HCl (pH-8). Replicates of each 384-sample pool were placed in the S4-flow cell according to the NovaSeq-XP workflow (Illumina, USA) and subjected to dual-indexed on a NovaSeq 6000 platform. The 141 samples of January 2021 were sequenced with single-end 35 pb read length while the 45 samples collected in July 2021 were sequenced with paired-end 150 pb read length.

Data analysis: the resulting sequence data were processed using DRAGEN COVID Lineage (v3.5.1) (Illumina, CA, USA) to obtain consensus sequences, Nextstrain clades (https://nextstrain.org/sars-cov-2, accessed on 31 August 2021), and Pangolin lineages (https://cov-lineages.org/pangolin.html, accessed on 31 August 2021) when at least 5 SARS-CoV-2 probes were detected.

Among them, samples covering at least 75% of the SARS-CoV-2 genome and 85% of the spike region were analyzed for alignment. Consensus sequences were aligned to the SARS-CoV-2 Wuhan-Hu-1 reference genome (NC_045512.2) and 109 GISAID sequences of Pangolin lineages detected in France using MAFFT (v.7.475). Maximum likelihood trees were then calculated from these alignments with IQTREE (v.2.0.3, evolutionary model: GTR + F + R3) [30] to confirm any clade and lineage results.

GISAID accession numbers are provided in the Supplementary Table S1. All the raw data are available on demand.

## 3. Results

### 3.1. Determination of the Clades and Lineages Based on Full-Length Genomes

Nasopharyngeal samples were taken early in the introduction of the Alpha and of the Delta variants to France (samples collected in January and July 2021, respectively). Patients were 33.7 ± 16.6 years old and most were females (105; 52.5%). Most (145/200; 72.5%) of the patients were symptomatic. Symptoms appeared the day before sampling in 55 patients, 2 to 4 days before in 67 patients, 5 to 7 days before in 15 patients, and 8 days or more before in 8 patients. Clades were identified by NextClade and lineages were determined using the PANGOLIN classification based on the analysis of full-length genome sequences (Table 2). In January 2021, three strains were more frequently detected: the Alpha variant that belonged to the clade 20I and the lineage B.1.1.7 (48/146, 32.9%), followed by 37 (25.3%) strains belonging to the clade 20A and the lineage B.1.160 and 37 variants belonging to the clade 20E(EU1) and the lineage B.1.177. Most of the strains collected in July (51/54, 95.6%) were Delta variants (clade 21A), with a majority of strains belonging to the lineage B.1.617.2 (*n* = 20) and AY.4 (*n* = 19).

### 3.2. Comparison of the Spike Sequences Obtained with the Illumina and Pacbio SMRT Systems

We developed a PacBio SMRT sequencing protocol that targeted the S1 domain and assessed its suitability for accurately predicting the lineage of a variant. First, we sequenced 146 samples collected in January 2021 with Illumina COVIDseq (with the recommended single-end 35 nt read length). Among them, 141/146 (96.6%) samples were successfully sequenced with PacBio SMRT sequencing. The first protocol (PacBio) produced a final sequence length of 2465 nt mapped on the reference genome from the positions 21358 to 23823. Therefore, 2271 nt overlapped the spike gene (the 757th first amino acids), which covered 59% of the S gene. The region encoding the S1 domain, including the RBD (22520–23186), was fully covered. The Illumina protocol generated the whole genome consensus.

**Figure 1:**
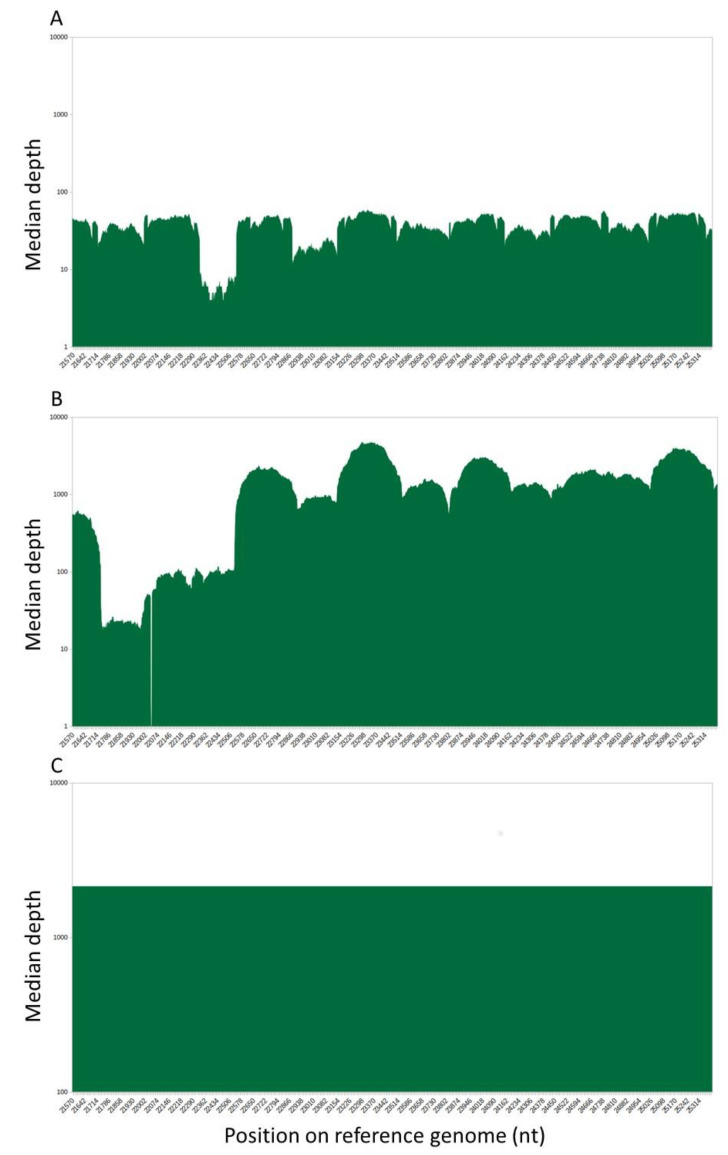
Sequencing depth over the spike gene with Illumina COVIDseq protocol. Logarithmic representation of the number of reads mapped on the reference genome, zoomed on the spike region. The x-axis was labeled from 21563 and 25384, corresponding to the positions on the complete genome. (**A**). Sequencing depth over the spike gene with Illumina COVIDseq protocol with singled-end 35 nt read-length sequencing and (**B**) paired-end 150 nt read-length sequencing. (**C**) Sequencing depth over the spike gene with PacBio protocol.

We then compared the S1 domain sequences obtained with the two platforms. The PacBio SMRT protocol identified mutations in 14/141 variants (9.9%) that were missed by the Illumina protocol (Table 3). All the missed mutations were due to a lack of coverage (<10 reads) and were grouped on the same amplicon NC_045512.2:22903–23122 (missing mutations: N440K, S477N, T478K, E484K, N501Y, A522S). A second amplicon, NC_045512:22346-22516 was less represented in the data, without consequences on the resulting mutation profiles (Figure 1A).

Consequently, for the sequencing of the 54 samples from July 2021, we extended the read length to 150 nt and used a paired-end kit; among these samples, 45/54 (83.3%) were sequenced successfully with PacBio SMRT system. The obtained depth of sequencing was greatly improved (Figure 1B), but we still missed some mutations in 13/45 (28.9%) samples (Table 3). Some mutations (*n* = 7) were not detected due to a default of amplification at the beginning of the spike gene (Figure 1B) while some others were completely absent of the variant calling results (*n* = 3), but most of them (*n* = 9) correspond to the presence of the mutation at less than 50% (not present in the resulting consensus sequence). The missing mutations were not located in the same region as previously, but rather over the amplicon NC_045512.2:21743–21961 (T95I, V130F, G142D, E156G, F157del, and R158del, A222V). It was noteworthy that as the PacBio protocol amplified only one long amplicon, the resulting coverage was always close to 100% when the amplification succeeded (with a median coverage of 2137 reads per position) (Figure 1C) but sequencing failed in 14/200 samples (7%). Taken together, the most frequently missed mutations were G142D (*n* = 11), E156G, 157del, 158del (*n* = 6), N501Y (*n* = 6), S477N (*n* = 5), T95I (*n* = 3), V130F (*n* = 2), and E484K (*n* = 2). 

Lastly, we checked the minority variants found by the two protocols, to assess the capacity of the PacBio SMRT system to retrieve diversity as accurately as the Illumina COVIDseq system, but with a longest PCR product. The same minority variants were detected with both protocols in three samples and the resulting percentages were quite similar (L5F: 46.4% with PacBio and 25.6% with Illumina; L176F: 22.6% with PacBio and 37.5% with Illumina; and 27.4% with PacBio and 31.2% with Illumina).

### 3.3. Determination of SARS-CoV-2 Lineage by S1 Domain Key Position Analysis

We developed an automated classification pipeline based on 70 S1 domain key mutations, characteristic of the major variants circulating in Europe from the beginning of the epidemic crisis to October 2021 (Table 1). The key mutations detected in the S1 domain were used to determine the SARS-CoV-2 lineage. For example, a variant harboring mutations S477N and D614G was identified as belonging to lineage B.1.160. To assess the suitability of this approach, we compared the lineages obtained with this pipeline with those obtained by analyzing full-length genomes.

Lineages were assigned for 172/186 samples (92.5%). The 14 samples whose lineage could not be determined included strains belonging to lineage B.1 (*n* = 5), B.1.241 (*n* = 3), B.1.1.317 (*n* = 2), B.1.2 (*n* = 1), B.1.1.28 (*n* = 1), B.1.1.186 (*n* = 1), and B.1.214 (*n* = 1) which were not included in the international variants monitoring (Table 4). The clade was then deduced according to the lineage (Table 1). For the variants belonging to the clade 21A, all were assigned to the lineage B.1.617.2, but we were unable to determine the AY sub-lineages. 

## 4. Discussion

The rapid emerging new SARS-CoV-2 variants must be accurately identified in order to follow-up their spread, particularly of any immune-escaping variants that might interfere with vaccination or monoclonal antibody (mAb) treatment. We developed a simple protocol for sequencing the region encoding the S1 domain using PacBio SMRT technology, because the emergence of such long-read technology has greatly facilitated the assembly of viral sequences. We also developed an automated pipeline for determining the SARS-CoV-2 variant lineages based on key amino acid positions. Analysis of the S1 domain, including the RBD, is well suited to monitor the appearance of immune-escaping mutations [31].

The sequences of the S1 domain of 186 samples obtained with the Sequel Platform (PacBio) were compared to those obtained by NovaSeq 6000 deep sequencing (Illumina). We found that the S1 domain was inadequately covered in the Illumina sequences, likely due to a less effective amplification for some of the designed ARTIC amplicons and the higher amount of material that can affect the read depths. This problem might be reduced or even suppressed with the use of the currently last available version (v4). In addition, the missing amplicons seems variant-dependent, with a difference of coverage between Delta and Alpha/B.1.160/B.1.177 variants. The full coverage of this region is critical since mutations contributing to virus escape from vaccine-induced neutralization are located in the S1 domain. For instance, the E484K mutation in the RBD notably described in the Beta and Gamma variants seems to confer resistance to the neutralizing capacity of fully vaccinated individuals [32,33]. The variant belonging to the lineage P.1 that emerged in Brazil has been responsible for reinfections, perhaps because of the three mutations in the RBD [34]. The variant belonging to lineage B.1.351 also resists neutralization due to mutations in the RBD and non-RBD domains [14,35]. Thus, the S1 domain must be fully covered in order to detect already known mutation conferring resistance to neutralizing antibodies and to identify new mutations (SNP, deletions, or insertions) in the RBD in new variants that are less sensitive to neutralizing antibodies. Sequencing the whole of the S1 domain is also of importance as this is the region targeted by monoclonal antibody therapies. Two neutralizing antibodies cocktails (bamlanivimab/etesevimab and casirivimab/imdevimab) have been authorized by the US Food and Drug Administration and the European Medicines Agency for treating patients at high risk of severe disease. Detailed knowledge of the region encoding the S1 domain is needed in order to identify the substitutions associated with reduced neutralizing antibody activity if treatment fails, since resistant mutants may emerge following the use of these neutralizing antibodies [24]. The variant belonging to the lineage B1.617.2 was shown to be resistant to the mAb bamlanivimab due to the escape mutation L452R [18] and was also less sensitive to sera from convalescent patients [36]. In addition, long reads data offer the opportunity to get the haplotypes and, thus, analyze whether the mutations detected are located on the same SARS-CoV-2 genome or on distinct genomes.

Lastly, we wanted to determine whether sequencing the S1 domain would enable us to determine accurately the virus lineage. The automated pipeline based on key mutations that are characteristic of some variants enabled us to assign clades to 172/186 (92.5%) sequences with a perfect agreement with the Illumina pipeline. The remaining unclassified variants belonged to less frequent variants circulating in Europe, which were not followed by the French monitoring system. The determination of the lineage is important because some are naturally less sensitive to neutralizing antibodies than are others. The lineage determination is also helpful to assess the current and future circulation of the different variants, like that of the strains belonging to the lineage B.1.1.7 and more recently that of the B.1.617.2 [37,38]. 

Our study has some limitations. Our pipeline does not allow the determination of the precise sub-lineage of Delta variant, because the differences detected in the S1 domain are not discriminant enough, with an exception for the AY.1 and AY.2 lineages, characterized by the K417N mutation [39]. Interestingly, K417N, L452R, E484K, and E484Q are the mutations known to disrupt receptor-binding domain (RBD) binding capacity, making them more infectious by immune escape against the current vaccines [40]. Another limitation is the design that was not a head-to-head comparison on consecutive clinical samples. Samples were selected based on successful sequencing by using the IlluminaCOVIDSeq approach. A total of 14/200 (7%) samples failed to sequence using PacBio SMRT, indicating the need of optimization, particularly for samples with low viral load. Further studies are alos needed to compare the two sequencing protocols.

## 5. Conclusions

We conclude that the PacBio SMRT technology is a suitable system for sequencing the SARS-CoV-2 spike protein S1 domain. It covers the whole domain, which is essential for virological follow-up, especially for monitoring the acquisition of mutations conferring full or partial resistance to neutralizing antibodies. This approach also offers the possibility to determine the lineage of the circulating variants by a partial sequencing.

## Figures and Tables

**Table 1:** Key mutations used for clade/lineage determination.

Clade	20I/ B.1.1.7	20I/ B.1.1.7	20I/ B.1.1.7	20H/ B.1.351	21A/ B.1.617.2	21A/ B.1.617.2	20J/ P.1	21H	20D/ C.36.3	20B/ B.1.1.318	20A/ B.1.620	19B/ A.27	19B/ A.28	20I/ B.1.1.7	20B/P.3	20A/ B.1.160	20E/ B.1.177	20A/ B.1.214.2	20A/ B.1.221	21C/ B.1.427	21D/ B.1.525	21F/ B.1.526	20C/ B.1.526.1	20C/ B.1.616	21B/ B.1.617.1	20A/ B.1.617.3	20A/ B.1.619	21G/ C.37
Lineage	B.1.1.7	B.1.1.7+ E484K	B.1.1.7+ E484Q	B.1.351	B.1.617.2	AY.1/AY.2	P.1	B.1.621	C.36.3	B.1.1.318	B.1.620	A.27	A.28	B.1.1.7+ L452R	B.1.1.28.3 /P.3	B.1.160	B.1.177	B.1.214.2	B.1.221	B.1.427/ B.1.429	B.1.525	B.1.526	B.1.526.1	B.1.616	B.1.617.1	B.1.617.3	B.1.619	C.37
WHO	Alpha	Alpha	Alpha	Beta	Delta	Delta	Gamma	Mu	N/A	N/A	N/A	N/A	N/A	N/A	N/A	N/A	N/A	N/A	N/A	Epsilon	Eta	Iota	N/A	N/A	Kappa	N/A	N/A	Lambda
L5F																						F						
P9L																												
S12F									F																			
S13I																				I								
L18F				(F)			F					F																
T19R					(R)	(R)																				(R)		
T20N							N																					
P26S							S				S																	
Q52R																					R							
H66D																								D				
A67V																					V							
69del	del	del	del						del		del		del	del							del							
70del	del	del	del						del		del		del	del							del							
G75V									V																			V
T76I																												I
D80A/G				A																			G					
T95I					(I)	(I)		I		I												I			(I)			
S98F																			F									
V126A											A																	
C136F																												
D138Y							Y																					
141del															del													
G142D/del/V					(D)	(D)									del									V		(D)		
143del															del													
144del/T	del	del	del					(T)		(del)	del			del							del		del	del				
Y145S/insN								(S)	(insN)																			
W152R/C									R											C								
E154K																									K *			
E156G					(G)	(G)																						
157del/S					(del)	(del)																	S			(del)		
158del					(del)	(del)																				(del)		
R190S							S																					
I210T																											T	
214insTDR																		insTDR *										
D215G				(G)																				G				
A222V																	V		(V)									
242del				(del)							(del)																	
243del				(del)							del				del													
244del				(del)							del				del													
H245Y											Y																	
247del																												del
248del																												del
249del																												del
250del																												del
251del																												del
252del																												del
D253G/del																						G						del
R346K/S								K	S																			
Q414K																		K										
K417N/T				(N)		N *	T																					
N440K																											K *	
Y449H																												
N450K																		K										
L452R/Q					R *	(R)			R *			R *		R *						R *			R *		R *	R *		Q *
S477N											N *					N						N^						
T478K					K *	(K)																						
V483A																								A				
E484K/Q		K *	Q *	K *			K *	(K)		K *	K *				K *						K *	K^			Q *	Q *	K *	
F490S																												S
N501Y/T	Y *	Y *	Y *	Y *			Y *	Y				Y *	T *	Y *	Y *													
A570D	D	D	D											D														
D614G	G	G	G	G	G	(G)	G	G		G	G			G	G	G	G	G	G	G	G	G	G	G	G	G	G	G
A653V												V																
H655Y							Y					Y	Y											Y *				
G669S																								S				
Q677H									H			(H)									H							
N679K																												
P681H/R	H	H	H		R	(R)		H		H *	H			H	H										R	R		
A701V				V																		V						
T716I	I	I	I											I				I										

*: Essential mutation to validate the profile. ^: At least one mutation required to validate the profile. (): Possible mutation, not required to validate the profile but improves discrimination. Only mutations detected in the S1 domain were covered by the fragment amplified by PacBio SMRT sequencing.

**Table 2:** Sample lineages and clades.

Clade	Lineage	Month of Collection		Total
		January	July	
20I	B.1.1.7	48	3	51
20A	B.1.160	37		37
20E(EU1)	B.1.177	37		37
21A	B.1.617.2		20	20
21A	AY.4		19	19
20A	B.1.221	8		8
20A	B.1	4		4
21A	AY.34		6	6
21A	AY.9		4	4
20B	B.1.1.241	3		3
20B	B.1.1.317	2		2
20H	B.1.351	2		2
20C	B.1	1		1
20B	B.1.1.186	1		1
20B	B.1.1.28	1		1
20G	B.1.2	1		1
20A	B.1.214	1		1
21A	AY.23		1	1
21A	AY.7.1		1	1
	Total	146	54	200

**Table 3:** List of mutations detected with the Illumina and PacBio SMRT systems in 14 patient samples giving discrepant results.

Sample	Clade	Lineage	Mutations Detected with Illumina	Mutations Detected with Pacbio
20	20A	B.1.214	T95I, D614G, T716I	T95I, **T478K**, D614G, T716I
33	20A	B.1.160	D614G	**S477N**, D614G
62	20A	B.1.160	D614G	**S477N,** D614G
79	20A	B.1.160	D614G	**S477N, A522S**, D614G
110	20A	B.1.160	D614G	**S477N**, D614G
111	20A	B.1.160	D614G	**S477N,** D614G
136	20B	B.1.1.186	L18F, D614G	L18F, **N440K,** D614G
30	20H (Beta)	B.1.351	L18F, D80A, D215G, 241del, 242del, 243del, K417N, N501Y, D614G, A701V	L18F, D80A, D215G, 241del, 242del, 243del, K417N, **E484K**, N501Y, D614G, A701V
134	20H (Beta)	B.1.351	D80A, D215G, 241del, 242del, 243del, K417N, D614G, A701V	D80A, D215G, 241del, 242del, 243del, K417N, **E484K, N501Y**, D614G, A701V
15	20I (Alpha)	B.1.1.7	69del, 70del, 144del, A570D, D614G, P681H, T716I	69del, 70del, 144del, **N501Y**, A570D, D614G, P681H, T716I
27	20I (Alpha)	B.1.1.7	69del, 70del, 144del, A570D, D614G, P681H, T716I	69del, 70del, 144del, **N501Y**, A570D, D614G, P681H, T716I
56	20I (Alpha)	B.1.1.7	69del, 70del, 144del, A570D, D614G, P681H, T716I	69del, 70del, 144del, **N501Y**, A570D, D614G, P681H, T716I
73	20I (Alpha)	B.1.1.7	69del, 70del, 144del, A570D, D614G, P681H, T716I	69del, 70del, 144del, **N501Y**, A570D, D614G, P681H, T716I
116	20I (Alpha)	B.1.1.7	69del, 70del, 144del, A570D, D614G, P681H, T716I	69del, 70del, 144del, **N501Y,** A570D, D614G, P681H, T716I
143	21A (Delta)	AY.4	T19R, E156G, 157del, 158del, L452R, T478K, D614G, P681R	T19R, **T95I, G142D**, E156G, 157del, 158del, L452R, T478K, D614G, P681R
153	21A (Delta)	AY.4	T19R, E156G, 157del, 158del, L452R, T478K, D614G, P681R, D950N	T19R, **G142D**, E156G, 157del, 158del, L452R, T478K, D614G, P681R
163	21A (Delta)	AY.4	T19R, L452R, T478K, D614G, P681R, D950N	T19R, **G142D, E156G, 157del, 158del**, L452R, T478K, D614G, P681R
167	21A (Delta)	AY.4	T19R, L452R, T478K, D614G, P681R, D950N	T19R, **G142D**, **E156G, 157del, 158del**, L452R, T478K, D614G, P681R
169	21A (Delta)	AY.4	T19R, E156G, 157del, 158del, L452R, T478K, D614G, P681R	T19R, **G142D**, E156G, 157del, 158del, L452R, T478K, D614G, P681R
170	21A (Delta)	AY.4	T19R, L452R, T478K, D614G, P681R	T19R, **G142D, E156G, 157del, 158del**, L452R, T478K, D614G, P681R
189	21A (Delta)	AY.4	T19R, E156G, 157del, 158del, L452R, T478K, D614G, P681R	T19R, **T95I**, **G142D**, E156G, 157del, 158del, L452R, T478K, D614G, P681R
186	21A (Delta)	AY.7.1	T19R, L452R, T478K, D614G, P681R	T19R, **E156G, 157del, 158del**, L452R, T478K, D614G, P681R
142	21A (Delta)	AY.9	T19R, E156G, 157del, 158del, A222V, L452R, T478K, D614G, P681R	T19R, **V130F,** E156G, 157del, 158del, A222V, L452R, T478K, D614G, P681R
183	21A (Delta)	AY.9	L452R, T478K, D614G, P681R, D950N	**T19R, V130F, G142D, E156G, 157del, 158del, A222V,** L452R, T478K, D614G, P681R
185	21A (Delta)	AY.9	T19R, E156G, 157del, 158del, A222V, L452R, T478K, D614G, P681R	T19R, **G142D**, E156G, 157del, 158del, A222V, L452R, T478K, D614G, P681R
157	21A (Delta)	AY.23	T19R, L452R, T478K, D614G, P681R, D950N, V1264L	T19R, **G142D**, **E156G, 157del, 158del**, L452R, T478K, D614G, P681R
154	21A (Delta)	AY.34	T19R, E156G, 157del, 158del, L452R, T478K, D614G, Q677H, P681R, D950N	T19R, **T95I**, **G142D**, E156G, 157del, 158del, L452R, T478K, D614G, Q677H, P681R

The mutations listed above were detected using the Wuhan-Hu-1genome sequence (NC_045512.2) as reference. In **bold**: mutations detected by the PacBio SMRT platform.

**Table 4:** Comparison of the lineages determined with full-length genomes and the S1-encoding region based on key positions.

Full Length Genome (Illumina)			Spike S1 Region (PacBio)	
Clade	Lineage	*n*	Lineage	*n*
20A	B.1.221	8	B.1.221	8
20A	B.1	4	NA	4
20A	B.1.214	1	NA	1
20A	B.1.160	37	B.1.160	37
20B	B.1.1.186	1	NA	1
20B	B.1.1.241	3	NA	3
20B	B.1.1.28	1	NA	1
20B	B.1.1.317	2	NA	2
20C	B.1	1	NA	1
20E (EU1)	B.1.177	37	B.1.177	37
20G	B.1.2	1	NA	1
20H	B.1.351	2	B.1.351	2
20I	B.1.1.7	45	B.1.1.7	45
21A	B.1.617.2	17	B.1.617.2	43
21A	AY.4	16		
21A	AY.34	4		
21A	AY.9	4		
21A	AY.23	1		
21A	AY.7.1	1		

NA: not assigned.

## Data Availability

Raw data are available on demand to the corresponding author: lhomme.s@chu-toulouse.fr.

## Supplementary Materials

The following are available online at https://www.mdpi.com/article/10.3390/v13122544/s1, Table S1: GISAID Accession Numbers.
